# A High-Throughput, Precipitating Colorimetric Sandwich ELISA Microarray for Shiga Toxins

**DOI:** 10.3390/toxins6061855

**Published:** 2014-06-11

**Authors:** Andrew Gehring, Xiaohua He, Pina Fratamico, Joseph Lee, Lori Bagi, Jeffrey Brewster, George Paoli, Yiping He, Yanping Xie, Craig Skinner, Charlie Barnett, Douglas Harris

**Affiliations:** 1 Molecular Characterization of Foodborne Pathogens Research Unit, United States Department of Agriculture-North Atlantic Area-Agricultural Research Service-Eastern Regional Research Center, Wyndmoor, PA 19038, USA; E-Mails: pina.fratamico@ars.usda.gov (P.F.); joe.lee@ars.usda.gov (J.L.); lori.bagi@ars.usda.gov (L.B.); jeffrey.brewster@ars.usda.gov (J.B.); george.paoli@ars.usda.gov (G.P.); yiping.he@ars.usda.gov (Y.H.); yanping.xie@ars.usda.gov (Y.X.); 2 Foodborne Toxin Detection and Prevention Research Unit, United States Department of Agriculture-Pacific West Area-Agricultural Research Service-Western Regional Research Center, Albany, CA 94710, USA; E-Mails: xiaohua.he@ars.usda.gov (X.H.); craig.skinner@ars.usda.gov (C.S.); 3 NanoDetection Technology, Inc., Franklin, OH 45005, USA; E-Mails: cbarnett1951@gmail.com (C.B.); dough@ndtbio.com (D.H.)

**Keywords:** B-PER, colorimetry, detection, ELISA, high-throughput, microarray STEC, microtiter plate, precipitating, toxin typing

## Abstract

Shiga toxins 1 and 2 (Stx1 and Stx2) from Shiga toxin-producing *E. coli* (STEC) bacteria were simultaneously detected with a newly developed, high-throughput antibody microarray platform. The proteinaceous toxins were immobilized and sandwiched between biorecognition elements (monoclonal antibodies) and pooled horseradish peroxidase (HRP)-conjugated monoclonal antibodies. Following the reaction of HRP with the precipitating chromogenic substrate (metal enhanced 3,3-diaminobenzidine tetrahydrochloride or DAB), the formation of a colored product was quantitatively measured with an inexpensive flatbed page scanner. The colorimetric ELISA microarray was demonstrated to detect Stx1 and Stx2 at levels as low as *~*4.5 ng/mL within *~*2 h of total assay time with a narrow linear dynamic range of *~*1–2 orders of magnitude and saturation levels well above background. Stx1 and/or Stx2 produced by various strains of STEC were also detected following the treatment of cultured cells with mitomycin C (a toxin-inducing antibiotic) and/or B-PER (a cell-disrupting, protein extraction reagent). Semi-quantitative detection of Shiga toxins was demonstrated to be sporadic among various STEC strains following incubation with mitomycin C; however, further reaction with B-PER generally resulted in the detection of or increased detection of Stx1, relative to Stx2, produced by STECs inoculated into either axenic broth culture or culture broth containing ground beef.

## 1. Introduction

In the United States alone, 31 major foodborne pathogens account for approximately 9.4 million illnesses, 56,000 hospitalizations and 1350 deaths per year [1]. Of those 31 pathogens, Shiga toxin-producing *E. coli* (STEC) O157 and non-O157 serogroups are estimated to be responsible for *~*176,000 domestically acquired food-borne infections annually, with non-O157 STEC accounting for approximately two-thirds of the infections [1,2]. Shiga toxins (Stx) produced by STEC are proteinaceous biomolecules with a molecular weight of *~*70 kDa, and they are encoded by lambda-like bacteriophages integrated into the bacterial chromosome. There are two main types of Stx, referred to as Stx1 and Stx2, that only share *~*55% amino acid sequence homology, and the LD_50_ for Stx2 is *~*400-times less than that for Stx1 [3]. The genetic variability of *stx* genes is well known, and many Stx1 and Stx2 subtypes exist. The Stx1 family has exhibited three major subtypes (Stx1a, Stx1c and Stx1d), which further divide into multiple genetic variants. The Stx2 family branches into seven major subtypes (Stx2a, Stx2b, Stx2c, Stx2d, Stx2e, Stx2f and stx2g), which further subdivide into a total of 93 genetic variants [4]. Stx1 is located in the periplasmic fraction of the cell, and its production is induced by low iron conditions [5]. Stx2 is found primarily in the extracellular fraction, and its production is induced by a number of antibiotics, including the chemotherapeutic agent, mitomycin C [5,6]. Hull *et al.* [7] demonstrated that the production of Stx1 was also increased with the addition of mitomycin by using an immunoblot colony assay for the detection of STEC in fecal samples. Antibiotics induce the “SOS response”, resulting in a switch from lysogeny to the lytic cycle and increased bacteriophage production [8]. This results in increased production and release of Shiga toxins.

There are multiple means for the relatively rapid characterization or “typing” of bacteria, including food-borne pathogens and/or the toxins or other virulence factors that they produce, using phenotyping and genotyping strategies [9]. If present in a high enough concentration, Stx may be detected and differentiated using specific antibodies in immunological assays. Combined with other genotyping techniques, such as pulsed-field gel electrophoresis or multilocus sequence typing, toxin typing may enhance the classification of bacteria in support of rapid food safety testing and/or epidemiological investigations [10]. 

Though similar to a recently described high-throughput detection platform [11,12], the colorimetric enzyme-linked immunosorbent assay (ELISA) microarray presented herein was developed using intact, 96-well, polystyrene microtiter plates. Employed as a toxin typing array, this technique is not only rapid, but also has exhibited sufficient sensitivity to detect Stx at the low nanogram per milliliter limit. Food producers and regulatory agencies may potentially employ this system to rapidly screen large numbers of food samples for toxins, as well as for other antigens (e.g., bacterial cells, cell fragments, metabolites, *etc.*), using the appropriate combination of antibodies.

## 2. Results and Discussion

### 2.1. Preliminary Assay Development

**Figure 1:**
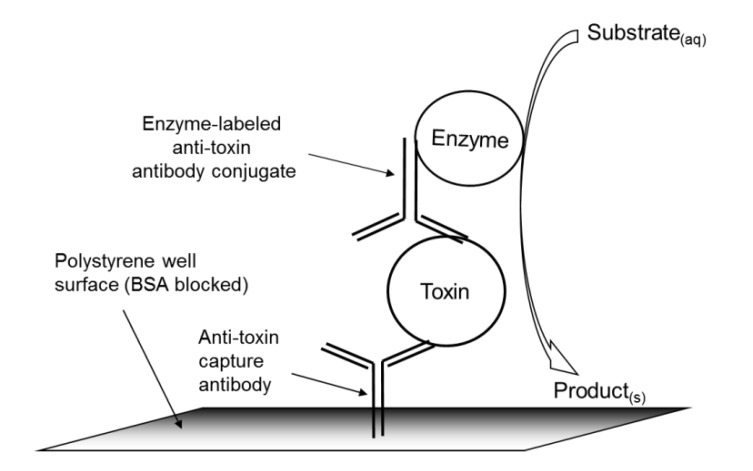
Schematic for colorimetric sandwich ELISA. Clear-walled, clear-bottomed polystyrene 96-well microtiter plates were array-printed with capture antibodies; the remainder of the polystyrene surface was blocked with BSA and the toxin sample introduced, and enzyme (HRP)-labeled antibody conjugate (reporter) was added to complete the sandwich. A flatbed scanner was used to generate images of the colored, enzymatic product precipitate and, hence, to detect captured toxins.

The initial effort in the generation of this colorimetric ELISA microarray (schematic representation displayed in Figure 1) was focused on the minimization of the “comet tailing” of arrayed features or spots, maximizing signal response, and the optimization of signal-to-noise ratio, while reducing error by increasing repeatability. Comet tailing has been observed to be the result of the unwanted binding of excess printed capture antibody outside of array spotted areas. In other words, saturation of adsorptive binding sites on the polystyrene surface leaves behind excess, unbound antibody that, for the most part, rinses away during subsequent washes. However, even though these washes are rapid (≤1 min), capture antibody appears to instantaneously adsorb to “virgin” polystyrene, resulting in asymmetrical spots. The capture of analyte at these asymmetrical regions leads to comet tailing that was prevented by the addition of blocker BSA to initial wash solutions (data not shown). Blocking with BSA is common practice in immunoassays, but here, BSA was also specifically employed to compete with unbound mAb for free adsorption sites on the polystyrene substrate. Furthermore, the binding of precipitated colorimetric development reagent (cleaved 3,3-diaminobenzidine tetrahydrochloride (DAB), the HRP substrate) to the polystyrene surface appeared to be enhanced by the presence of proteinaceous blocking reagents (data not shown).

**Figure 2:**
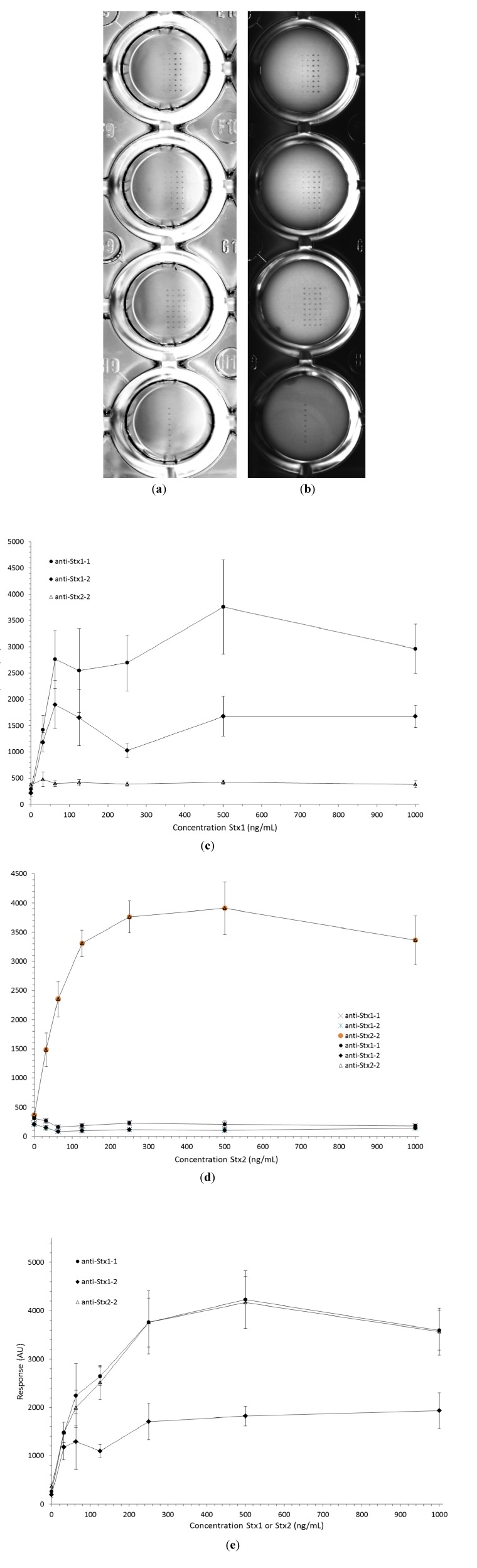
Standard curve for colorimetric sandwich ELISA microarray detection of Stx1 and/or Stx2. Well bottoms of a 96-well microtiter plate were contact printed (eight replicates) from left to right with an HRP-antibody marker (Column 1), two different monoclonal antibodies (mAbs) to Stx1 (1-2 in Column 2 and 1-1 in Column 3) and a mAb raised against Stx2 (2-2 in Column 4). Serial dilutions of Stx1, Stx2 and Stx1 mixed with Stx2 were reacted and visualized with the addition of a cocktail of the mAbs (HRP-labeled) followed by colorimetric development with an HRP-precipitating substrate. A representative sample flatbed scanned image of Stx1 and Stx2 detection at 125, 62, 31 and 0 ng/mL (top to bottom) before (**a**) and after (**b**) the addition of diluted (1:3 in distilled water) skin lotion. The standard curves for the detection of Stx1 (**c**); Stx2 (**d**); and Stx1 mixed with Stx2 (**e**).

Typical scans through the bottom of the top-side up microtiter plates yielded an unanticipated light-to-dark gradient over the surface of individual wells (refer to Figure 2a). The concern of this phenomenon affecting signal-to-noise ratios led to testing opaque liquids (e.g., half and half dairy product, mayonnaise, skin lotion, *etc.*), about half filling microtiter plate wells, for their ability to limit the focal depth and subsequent sidewall reflections/shadows, thus minimizing the modification of signal intensity and creating a uniform background. Figure 2b indeed exhibits a marked uniformity in background response for scans of individual wells; however, it was determined that error (σ) associated with the mean signal-to-noise ratio was not significantly improved by the addition of an opaque liquid to the plate wells prior to scanning (data not shown). This was most likely a result of the signal and background acquisition technique, where the background was a concentric, “local” ring surrounding the signal area.

### 2.2. Stx Standard Curves with the Colorimetric ELISA Microarray Platform

Figure 2a shows the results of a typical image obtained from scanning an enzymatically-developed ELISA microarray plate (with the enzyme solution removed). Each column within individual wells contains eight replicated spots of microarrayed capture antibody or HRP-labeled antibody marker. Two-fold serially diluted Stx1, Stx2 or a mixture of Stx1 and Stx2 were subjected to the colorimetric ELISA detection assay as described. Figure 2c–e shows standard curves for the Shiga toxins indicating a narrow dynamic range of 1–2 orders of magnitude, a saturation of response around 75–125 ng/mL and an apparent limit of detection (LOD) of *~*32 ng/mL. Note that the source of Stx used was *~*50% pure [13], so all of the concentrations were actually half of that reported. Therefore, the actual LOD of Stx was *~*16 ng/mL. In-house analysis suggested that the commercially purchased Stx1 and Stx2 were only *~*14% pure (data not shown), thus reducing the apparent LOD further to *~*4.5 ng/mL. Using the assay parameters presented herein, additional experimentation revealed no detection response for Stx1 (or Stx2) at concentrations of *~*≤2 ng/mL (based upon a 14% purity-adjusted concentration) (data not shown).

The dip in response for Stx1 as detected by anti-Stx1-2 (Figure 2c) was not real, but was due to a determinate array contact printing error (*i.e*., most likely poor capillary flow due to a transient dust particle) that resulted in severely low printing of all technical replicates for that concentration level. The saturation of response, either due to occupation by Stx analyte at all capture antibody binding sites or, more likely, saturation of the reaction site with the precipitated enzymatic product, was observed to be to a similar extent after both 5 and 60 min of enzymatic reaction. However, a slightly higher error (σ) in the net response was observed at 5 min relative to 60 min of development. Regardless, as with other precipitation-based enzymatic assays (e.g., dot immunoblots), the dynamic range typically suffers, resulting in a ~2 orders of magnitude linear dynamic range for various antigens [14,15,16,17]. There appeared to be no remarkable inhibition during the mutual detection of Stx1 and Stx2 (Figure 2e). Specificity was high for the mAbs, since no cross-reaction was observed (Figure 2d), and anti-Stx1-2 mAb had a significantly lower titer than anti-Stx1-1 (Figure 2e). This lack of cross-reaction also suggests that it was unlikely that there were any contaminants in the partially purified Stx standards that may have yielded false positive responses. Finally, a remarkable “hook effect” was observed at extremely high concentrations of Stx (*~*2000 ng/mL), most likely attributable to a distal site binding-induced conformational change that may cause the release of the (captured) antibody bond [18] (data not shown).

### 2.3. Colorimetric ELISA Microarray Detection of Stx Generated from STEC Enrichment Cultures

With standard curves for Stx1 and/or Stx2 established, the experimentation focus was shifted to investigating the detection of Stx produced by culture-enriched STECs. STECs were reported to not often produce Stx, even in mixed culture, unless induced by a foreign agent, such as the antibiotic, ofloxacin [19]. In this report, the induction by mitomycin C was studied, as well as the potential release of any cell-associated Stx by subsequent reaction with the protein extraction reagent, B-PER (the commercial reagent, B-PER, came pre-buffered in phosphate; so, corresponding controls were adjusted to the same reaction volumes with PBS, as indicated). 

Table 1 exhibits the semi-quantitative responses for the colorimetric ELISA microarray detection of Stx produced by overnight-cultured STEC, as well as non-Shiga toxin-producing or negative control *E. coli* and uninoculated ground beef. Table 1a shows that two of six strains with the potential to make Stx2 produce detectable levels of the toxin when grown overnight on TSB containing casamino acids. The addition of mitomycin C resulted in the induction of Stx2 in that five of the six strains produced detectable levels of Stx2. Note that considerable drops in the final, stationary phase cell concentration were observed for all the strains cultured in the presence of the antibiotic. The addition of B-PER to cells treated with mitomycin C elicited more positive immunological reactions, indicating that some Stx was cell-associated (Table 1b). Finally, a comparison of Table 1b and Table 1c suggests no major improvement on the release of cell-associated Stx from the constant mixing of B-PER with cells, as opposed to static incubation. This result suggested that the offline reaction between cells and B-PER was not necessary, so that subsequent B-PER reactions were conducted by the direct addition of the reagent to samples contained in the microtiter plates; and no further sample agitation was performed. Overall, Stx1 detection remarkably increased for enrichment cultures containing mitomycin C, and Stx1 was detected in even more strains upon the treatment of cultured cells with B-PER.

**Table 1:** The colorimetric ELISA microarray detection of Stx produced by enriched axenic broth cultures of STECs. Various strains of STECs and non-STEC controls were cultured overnight in TSB ± mitomycin C to observe the antibiotic induction of toxin formation, as detected by a sandwich ELISA microarray platform with colorimetric detection. B-PER, added to a portion of aliquots from overnight cultures, was statically incubated or shaken during incubation, and samples were tested for the release of cell-associated toxin. The stationary or final cell concentration was determined via total aerobic plate culture with TSA. (The mitomycin C concentration was 50 ng/mL; the capture antibody was 1:2 diluted; Color development reactions were for 60 min prior to scanning.) Legend: (?) questionable Shiga toxin detection (changed to “+” when allowed to react overnight); (−) no discernable detection; (+) low detection response; (++) moderate detection response; (+++) high detection response.

Strain	Serotype		−Mitomycin C							+Mitomycin C							
			Anti-Stx monoclonal antibody					Final cell concentration (CFU/mL)		Anti-Stx monoclonal antibody						Final cell concentration (CFU/mL)	
			1-1		1-2		2-2			1-1			1-2		2-2		
(**a**) Colorimetric ELISA microarray detection responses for enriched axenic broth cultures																	
(Stx1 producing)																	
05-6545	O45:H2		−		−		−	1.26 × 10^9^		?			−		−	8.90 × 10^8^	
96-3285	O45:H2		−		−		−	1.10 × 10^9^		−			+		−	1.78 × 10^8^	
TB352	O26:NM		−		−		−	7.10 × 10^8^		−			−		−	2.21 × 10^8^	
(Stx2)																	
08023	O121:H19		−		−		+	1.79 × 10^9^		−			−		+	2.54 × 10^8^	
94-0941	O145:H−		−		−		−	1.60 × 10^9^		−			−		−	7.20 × 10^8^	
94-0961	O111:H−		−		−		−	9.00 × 10^8^		−			−		+	4.10 × 10^8^	
96-1585	O121:H19		−		−		+	1.76 × 10^9^		−			−		+	3.39 × 10^8^	
(Stx1 and 2)																	
30-2C4	O157:H7		−		−		?	1.33 × 10^9^		−			+		+	7.00 × 10^8^	
SJ2	O26:H11		−		−		−	1.36 × 10^9^		−			−		+	5.65 × 10^7^	
(non-Stx producing controls)																	
B6-914	O157:H7		−		−		−	1.06 × 10^9^		−			−		−	1.72 × 10^8^	
K12	n/a		−		−		−	4.55 × 10^8^		−			−		−	2.16 × 10^8^	
(**b**) Colorimetric ELISA microarray detection responses for enriched axenic broth cultures subsequently treated with B-PER (static reaction)																	
(Stx1)																	
05-6545	O45:H2	−		−		−			1.26 × 10^9^		−	+		−		8.90 × 10^8^	
96-3285	O45:H2	−		−		−			1.10 × 10^9^		+	+		−		1.78 × 10^8^	
TB352	O26:NM	−		−		−			7.10 × 10^8^		−	−		−		2.21 × 10^8^	
(Stx2)																	
08023	O121:H19	−		−		+			1.79 × 10^9^		−	−		++		2.54 × 10^8^	
94-0941	O145:H−	−		−		−			1.60 × 10^9^		−	−		−		7.20 × 10^8^	
94-0961	O111:H−	−		−		−			9.00 × 10^8^		−	−		++		4.10 × 10^8^	
96-1585	O121:H19	−		−		−			1.76 × 10^9^		−	−		++		3.39 × 10^8^	
(Stx1 and 2)																	
30-2C4	O157:H7	−		−		−			1.33 × 10^9^		−	+		+		7.00 × 10^8^	
SJ2	O26:H11	−		−		?			1.36 × 10^9^		+	+		+		5.65 × 10^7^	
(non-Stx producing controls)																	
B6-914	O157:H7	−		−		−			1.06 × 10^9^		−	−		−		1.72 × 10^8^	
K12	n/a	−		−		−			4.55 × 10^8^		−	−		−		2.16 × 10^8^	
(**c**) Colorimetric ELISA microarray detection responses for enriched axenic broth cultures subsequently treated with B-PER (with shaking during reaction)																	
(Stx1)																	
05-6545	O45:H2	−		−		−			1.26 × 10^9^		−	+		−		8.90 × 10^8^	
96-3285	O45:H2	−		−		−			1.10 × 10^9^		++	++		−		1.78 × 10^8^	
TB352	O26:NM	−		−		−			7.10 × 10^8^		−	−		−		2.21 × 10^8^	
(Stx2)																	
08023	O121:H19	−		−		+			1.79 × 10^9^		−	−		+		2.54 × 10^8^	
94-0941	O145:H−	−		−		−			1.60 × 10^9^		−	−		−		7.20 × 10^8^	
94-0961	O111:H−	−		−		−			9.00 × 10^8^		−	−		+		4.10 × 10^8^	
96-1585	O121:H19	−		−		+			1.76 × 10^9^		−	−		+		3.39 × 10^8^	
(Stx1 and 2)																	
30-2C4	O157:H7	−		−		−			1.33 × 10^9^		+	+		+		7.00 × 10^8^	
SJ2	O26:H11	−		−		−			1.36 × 10^9^		+	+		+		5.65 × 10^7^	
(non-Stx controls)																	
B6-914	O157:H7	−		−		−			1.06 × 10^9^		−	−		−		1.72 × 10^8^	
K12	n/a	−		−		−			4.55 × 10^8^		−	−		−		2.16 × 10^8^	

### 2.4. Colorimetric ELISA Microarray Detection of Stx in STEC-Inoculated Ground Beef Enrichments

Table 2 exhibits the semi-quantitative responses for colorimetric ELISA microarray detection of Stx produced by STEC, as well as non-Shiga toxin-producing or negative control bacteria, cultured overnight in ground beef. Similar to the results displayed in Table 1a, mitomycin C appeared to induce the production of Stx1, though not as dramatically with the ground beef cultures. This observation was particularly noticeable for ground beef cultures not further treated with B-PER. Also comparable to the axenic culture study (Table 1a–c), the addition of B-PER was observed to greatly enhance the yield of immunologically detected Stx1 (Table 2a) relative to Stx2, providing additional evidence that Stx1 is more cell-associated than Stx2. Finally, expected Stx production was detected for all strains following B-PER treatment, whereas overall mitomycin C induction was rather limited for the ground beef-cultured STECs (Table 2a *versus* 2b).

**Table 2:** Colorimetric ELISA microarray detection of Stx produced by enriched broth cultures of STECs containing ground beef. Various strains of STECs and non-STEC controls were cultured overnight in TSB ± mitomycin C to observe the antibiotic induction of toxin formation as detected by a sandwich ELISA microarray platform with colorimetric detection. B-PER, added to a portion of aliquots from overnight cultures, was directly added to overnight cell culture aliquots held in the microtiter plate wells and statically incubated to promote the release of cell-associated toxin. (Inocula were 77 ± 40 CFU/mL; capture antibody was 1:10 diluted; the mitomycin C concentration was 66 ng/mL; color development reactions were for 60 min prior to scanning.) Legend: (?) questionable Shiga toxin detection (changed to “+” when allowed to react overnight); (−) no discernable detection; (+) low detection response; (++) moderate detection response; (+++) high detection response.

**Strain**	**Serotype**	**−Mitomycin C**			**−Mitomycin C; +B-PER**		
		**Anti-Stx monoclonal antibody**			**Anti-Stx monoclonal antibody**		
		**1-1**	**1-2**	**2-2**	**1-1**	**1-2**	**2-2**
(**a**) Colorimetric ELISA microarray detection responses for enriched ground beef subsequently treated with B-PER							
(Stx1 producing)							
00971	O26:H11	−	−	−	+	+	−
04162	O103:H8	−	−	−	+++	+++	−
98-8338	O111:NM	+	+	−	++	++	−
(Stx2)							
08-023	O121:H19	−	−	++	−	−	+++
94-0961	O111:H−	−	−	?	−	−	+
96-1585	O121:H19	−	−	++	−	−	+++
(Stx1 and 2)							
00-4748	O111:NM	−	−	+	+	+	+
30-2C4	O157:H7	−	−	++	+++	+++	++
SJ2	O26:H11	−	−	+	++	+++	+
(non-Stx producing controls)							
B6-914	O157:H7	−	−	−	−	−	−
K12	n/a	−	−	−	−	−	−
Ground beef	n/a	−	−	−	−	−	−
**Strain**	**Serotype**	**+Mitomycin C**			**+Mitomycin C; +B-PER**		
		**Anti-Stx monoclonal antibody**			**Anti-Stx monoclonal antibody**		
		**1-1**	**1-2**	**2-2**	**1-1**	**1-2**	**2-2**
(**b**) Colorimetric ELISA microarray detection responses for enriched ground beef containing mitomycin C and subsequently treated with B-PER							
(Stx1)							
00971	O26:H11	−	−	−	+	+	−
04162	O103:H8	−	−	−	++	++	−
98-8338	O111:NM	+	+	−	+++	+++	−
(Stx2)							
08-023	O121:H19	−	−	+++	−	−	++
94-0961	O111:H−	−	−	+	−	−	+
96-1585	O121:H19	−	−	+++	−	−	+++
(Stx1 and 2)							
00-4748	O111:NM	−	−	−	?	?	?
30-2C4	O157:H7	+	+	+++	+++	+++	+++
SJ2	O26:H11	+	+	+++	+++	+++	+++
(non-Stx controls)							
B6-914	O157:H7	−	−	−	−	−	−
K12	n/a	−	−	−	−	−	−
Ground beef	n/a	−	−	−	−	−	−

## 3. Experimental Section

### 3.1. Materials

Bovine serum albumin (BSA; Fraction V), glycerol, mitomycin C, phosphate-buffered saline (PBS; 10 mM phosphate, 2.7 mM KCl, 137 mM NaCl, pH 7.4) tablets and Tween 20 were obtained from Sigma (St. Louis, MO, USA). Partially purified Stx1 and Stx2 were from Toxin Technology, Inc. (Sarasota, FL, USA). The microarray “source” plates used were MicroAmp^®^ 384-conical well, polypropylene reaction plates (PE Biosystems, Carlsbad, CA, USA). Antibodies were printed onto the well bottoms of clear-walled, clear/transparent and flat-bottomed, polystyrene, 96-multiwell MICROLON 600 microtiter plates with high binding surfaces (Greiner Bio-One North America Inc., Monroe, NC, USA), which served as “destination” plates. Two monoclonal antibodies (mAbs) to Stx1, designated anti-Stx mAb 1-1 and 1-2 [20], and two mAbs to Stx2, designated anti-Stx mAb 2-1 and 2-2, were generated at the USDA Agricultural Research Service’s Western Regional Research Center (Albany, CA, USA), as previously described [21]. Two sets of the same batch of anti-Stx mAbs were pooled and conjugated with horseradish peroxidase (HRP) using a Lightning-Link HRP kit (Innova Biosciences, Cambridge, UK) according to the kit instructions. Set 1 contained 0.6 mg each of anti-Stx1-1, 2-1 and 2-2 and 0.4 mg of anti-Stx1-2 conjugated, desalted, concentrated and then diluted with 50% glycerol in water to *~*2.2 mg/mL. Set 2 contained 0.7 mg each of anti-Stx1-1, 2-1 and 2-2 and 0.075 mg of anti-Stx1-2 pooled, conjugated and then further diluted with 50% glycerol in water to a final concentration of *~*0.80 mg/mL. The Set 1 conjugate cocktail was employed for the axenic broth culture study presented in Table 1, and the Set 2 conjugate cocktail was employed for the Stx standard curve (Figure 2) and beef enrichment study (Table 2). Buffered protein extraction reagent (B-PER) in phosphate buffer, metal enhanced 3,3-diaminobenzidine tetrahydrochloride (DAB) and HRP-conjugated mouse anti-rabbit IgG (H + L), used as a microarray ELISA colorimetric internal control and marker, were from Pierce/Thermo Fisher Scientific (Rockford, IL, USA). *E. coli* O157:H7 strains were obtained from in-house stocks, except for *E. coli* K12 (ATCC 29425), which was obtained from the American Type Culture Collection (Manassas, VA, USA). Tryptic soy agar (TSA) and tryptic soy broth (TSB) were from Becton Dickinson (Sparks, MD, USA). Casamino acids were from Acumedia (Neogen Corp., Lansing, MI, USA). Any chemicals not mentioned were at least of reagent grade.

### 3.2. Apparatus

Antibody solutions were printed into 96-well microtiter plate wells using a Gene Machine Omnigrid Accent (Bucher, Basel, Switzerland) and a single SMP3 printing pin (TeleChem International, Inc., Sunnyvale, CA, USA). Colorimetric scans of the microarrayed microtiter plates were acquired with a Spotware Colorimetric Microarray scanner (Arrayit Corp., Sunnyvale, CA, USA). Centrifugation of microtiter plates was conducted in an Eppendorf model 5810R refrigerated centrifuge outfitted with an A-4-62 swinging bucket rotor (Eppendorf AG, Hamburg, Germany).

### 3.3. Enrichment Growth of E. coli

For axenic broth cultures (Table 1), a colony of each bacterial strain used was transferred to separate tubes containing 25 mL TSB, 10 g/L casamino acids and ± mitomycin C (50 ng/mL) and cultured for 18 h at 42 °C without mixing.

For enrichment cultures containing ground beef (Table 2), an overnight axenic culture was serially diluted into buffered peptone water, and an appropriate dilution was used to inoculate 8.3 g of ground beef [22]. Inoculum levels, 77 ± 40 CFU/mL, were later determined via spread plating 0.1 mL of diluted cultures onto TSA and incubating overnight at 37 °C. Inoculated ground beef samples were enriched at 42 °C for 18–20 h without mixing in separate filtered stomacher bags containing 25 mL TSB, 10 g/L casamino acids and ± mitomycin C (66 ng/mL).

### 3.4. Antibody Preparation and Microarray Printing

All mAbs and the HRP-labeled mAb conjugate “cocktail” adjusted to 50% glycerol were maintained at either 4 °C (mAbs) or −20 °C (cocktail). Immediately prior to use, mAbs or the cocktail was diluted (as indicated) in PBS containing 10% glycerol for either array printing or for use in the immunoassay. The relatively high concentration of glycerol was maintained in order to prevent the evaporation of the droplets and to maintain a hydrated state for the capture antibodies [23].

Approximately 30 μL of thoroughly-mixed capture antibody solutions, either 1:2 (axenic broth culture study) or 1:10 (inoculated ground beef culture study) diluted (in PBS containing 10% glycerol), were pipetted into separate wells of MicroAmp source plates. In order to remove any air bubbles, the plates were centrifuged at 1000 rpm (200 × *g*) for 2 min immediately prior to placing onto the microarray printer thermal block (maintained at 4 °C) and printing. Array printing was performed using the following settings: preprints/blots = 10; contact time = 0; dip and print velocity = 2 cm/s; dip and print acceleration = 10 cm/s^2^; with an SMP3 pin, which delivered a volume of *~*0.7 nL that produced spots of *~*100 μm in diameter per contact stroke. The pins were manually sonicated for 5 min in distilled H_2_O after each daily printing routine. Columns of 8 spots per each antibody were printed with a spot separation, from edge-to-edge, of 400 μm in both the “X-axis” and “Y-axis” directions. After printing, all wells were visually examined to ensure that spots were uniformly printed. Upon completion of printing, the spotted destination plates sat at RT in a constant “humidity chamber” (*~*60% RH) for 1 h at 4 °C prior to use.

### 3.5. Antibody Microarray Detection of Shiga Toxin in Multiwell Plates

The procedure for conducting a precipitating colorimetric ELISA immunoassay in the multiwell antibody microarray for the detection of Stx generally followed the one previously described for bacterial detection [24] with several modifications. All immunoassay procedures and reagents were at RT. Wells of the destination plate, preprinted with capture antibody, were washed with 200 µL of 1% BSA in PBS to reduce comet tailing. All wells were filled with 200 µL of PBST (PBS containing 0.05% Tween 20), immediately emptied by rapidly inverting the plate, and residual liquid was removed by striking the inverted plate onto an absorbent paper towel on the lab bench. This wash procedure was repeated. The plate wells were blocked with 200 µL of 1% BSA in PBS for 30 min. The BSA solution was removed, and the plate was washed as above. Analyte (100 µL for Figure 2 Stx standard curve generation or 200 µL, total, of cultured sample aliquots plus B-PER added 1:1 used in Table 1b,c and Table 2a,b) was then added and statically held at RT for 60 min. For the axenic culture experiment (Table 1b,c), the analyte was combined with B-PER and reacted statically or with shaking in separate 1.5-mL polypropylene microcentrifuge tubes for 60 min prior to reaction with capture antibodies. For the ground beef culture experiment (Table 2a,b), enrichment culture aliquots were combined with B-PER directly in the microtiter plate wells followed by static reaction, as indicated. The wells were washed twice with PBST, and excess liquid was removed as above. Next, 100 μL of HRP-mAb conjugate cocktail diluted 1:50 in PBS was added to each well followed by static incubation for 1 h at RT. Wells were washed (as above) twice with PBST and then once with PBS. To each well, 100 µL of freshly prepared (per the kit instructions) DAB was added and allowed to statically react at RT for 60 min (or as indicated). Excess DAB solution was removed via rapid inversion of the microtiter plate and gentle tapping of the inverted plate onto absorbent paper towels prior to most scans. A schematic representation of the immunoassay is displayed in Figure 1.

### 3.6. Microarray Scanning and Analysis

Microtiter plates were scanned from the bottom up using a Spotware Colorimetric Microarray scanner from Arrayit (Sunnyvale, CA, USA) following 60 min of colorimetric development. The scan settings used were 1200 dpi and 16-bit grayscale. Resultant scanned images (*.tiff files) were further analyzed using Array-Pro Analyzer software (version 4.5.1.73) [25]. Each well, which contained 8 printed spots per mAb, was considered an experimental unit. All experiments included 4 replicate wells per analyte or analyte concentration, thus resulting in 32 capture antibody spots in total. Raw, visible signal intensities corresponding to arrayed sample spots (whole “cell” areas without normalization) were obtained using an 8 × 3 array measurement grid of circles of identical size (10 pixels × 10 pixels, width × height). Background signal intensities were obtained using a “local ring” (a concentric circle) atop corresponding sample spots. (Measurement parameters for the local ring were: width = 2, erode = 2, filter = 1 and offset = 0.) Net spot intensities (raw sample responses minus corresponding to “local ring” median background responses) were compared, and the 2 highest and 2 lowest values were discarded. Net intensities were then averaged, and standard deviations were computed for the means reported in arbitrary units, or AUs.

## 4. Conclusions

This article documents the development of a novel high-throughput, multiplex method that combines a precipitating colorimetric ELISA in microarray format with inexpensive flatbed scanning for detection. The sandwich ELISA employed was successfully implemented in a microarray platform based on polystyrene, 96-well microtiter plate substrates, which further reduced the assay expense. The detection of Shiga toxins produced by various STEC strains was demonstrated with the colorimetric ELISA microarray platform. The lowest level of Stx1 or Stx2 detected in a total assay time of *~*1 to 2 h (5 or 60 min colorimetric development reaction with DAB, respectively) was *~*4.5 ng/mL, whether the analytes were separate or combined. Array analysis grids were manually positioned for data signal/noise analysis, a process that is more challenging at response levels for the lowest concentrations tested, since they were somewhat faint. Monoclonal antibodies (mAbs) were employed in this example, and as expected, immunoassays generated by other groups using polyclonal antibodies and/or more sensitive, though more expensive, luminescence detection fared better, with reported detection limits at the pg/mL level [26,27,28].

Commercial tests are typically qualified for sensitivity, as compared with other “gold standard” methods. Yet, most reports revealed the performance of several such tests with respect to the concentration of STEC cell, not the Stx detected. The developed microarray, ELISA LOD for Stx, of ~4.5 ng/mL, compares favorably to that of the 1 ng/mL LOD for VTEC-RPLA as reported by the manufacturer. It is expected that other, single-test, commercially available Stx detection kits (e.g., ImmunoCard STAT! EHEC, Premier EHEC and ProSpecT Shiga Toxin *E. coli* Microplate) have comparable LODs. While the reverse passive latex agglutination and immunochromatographic strip assays are typically fast (~10 min total assay time), the presented microarray ELISA assay time was more comparable to that of the typical microplate-based ELISAs represented by the latter two tests. The specificity of these tests have also been reported, but the typical focus was on Stx1 *vs.* Stx2, though subtypes have also been investigated [29,30]. Our focus was not on the specificity, though research in elucidating the specificity of the tested mAbs is ongoing. However, it is improbable that immunoassay-based methods will ever be able to specifically differentiate all of the genetic variants of the Stx subtypes, even though the microarray ELISA platform can readily accommodate the multiplexed detection of all known variants. This is because biorecognition elements (e.g., antibodies or aptamers) have yet to be developed and/or may never exhibit that level of specificity. Hence, microarray ELISA may only be applicable to differentiating subtypes at best. Though immunological methods may be employed to differentiate nucleic acids, genetic methods (hybridization or sequencing) will most likely be more effective at this task. This is especially true if a genetic variation does not result in a primary structure change for the Stx variant(s). The colorimetric ELISA microarray was further employed to detect Stx produced by STEC inoculated into either axenic broth culture or cultured ground beef. Though some Stx was detected in cultured samples, additional Stx (Stx1 more so than Stx2) was detected, particularly in axenic culture, upon induction with mitomycin C. In addition, Stx1 was more readily detected in B-PER-reacted culture samples, whereas Stx2 was often present even without B-PER treatment. This result provides further evidence that Stx1 is intrinsically more cell-associated than Stx2, as previously reported [5]. As for the limited influence of antibiotic on Stx production observed for STECs enriched in ground beef containing mitomycin C, it was possible that either the presence of the beef matrix and/or background flora inhibited induction. Quorum-sensing by co-cultured bacteria has indeed been previously implicated as a reason for the suppression of Stx production by STECs in mixed culture [19]. However, the varying responses for the mAbs, which have yet to be fully characterized with respect to Stx subtype recognition, was through no apparent defect of the developed assay or mAbs employed; the Stx subtypes or variants produced by the tested STEC strains may have been outside the specificity for the mAbs employed in this study.

Given that the developed assay signal keys on the accumulation of the precipitated enzymatic product, the saturation of the biorecognition site is typical and was most likely responsible for the seemingly narrow dynamic range of one to two orders of magnitude. Therefore, the assay may be quantitative, but over a small detection window of analyte concentration. The results suggest that the assay is best suited for testing conditions with either semi-quantitative or binomial detection. Though 60 min was used for the enzyme-based colorimetric development reaction, the same detection limit was observed with only 5 min of development, though the error (σ) associated with the signal-to-noise ratio was slightly higher (the average coefficient of variance increased *~*5%–10%), perhaps since scans were made with enzyme substrate solution still contained in the wells of the microtiter plate. With the appropriate antibodies or other biorecognition elements, this procedure may be used to rapidly screen large numbers of clinical or food samples for the presence of pathogens other than STECs.
